# Oxygen uptake of rainbow trout during acute warming: effects of oxygen supersaturation and aerobic exercise

**DOI:** 10.1242/jeb.252245

**Published:** 2026-08-07

**Authors:** Imogen G. Bellinger, Heather Bauer Reid, Graham D. Raby

**Affiliations:** ^1^ Department of Biology, Trent University, Peterborough, ON, Canada, K9L 1Z8; ^2^ Environmental and Life Sciences Graduate Program, Trent University, Peterborough, ON, Canada, K9L 0G2

**Keywords:** Climate change, Oxygen limitation, Swim tunnel, OCLTT, Respirometry

## Abstract

Naturally occurring oxygen supersaturation (hyperoxia) could provide a refuge for fish from heat waves if thermal tolerance is limited by the ability to take up sufficient oxygen to meet cellular oxygen demands during acute heat stress, as previously proposed. While several studies have assessed warming tolerance (CT_max_) in water breathers under hyperoxia, none have assessed whether warming tolerance during aerobic exercise (CTS_max_) is affected by hyperoxia. Here, we assessed whether hyperoxia could increase the thermal tolerance and metabolic rate of rainbow trout (*Oncorhynchus mykiss*) both at high swimming speeds (CTS_max_; 85% of maximum swimming speed) and in traditional CT_max_ trials. To do so, *n*=16 rainbow trout were subjected to thermal tolerance trials under two treatments: hyperoxia [150% dissolved oxygen (DO)] and normoxia (100% DO). For these trials, fish were warmed at a rate of 2°C h^−1^, with estimates of oxygen consumption (*Ṁ*_O_2__) generated for each 30 min (1°C) increment. Hyperoxia caused an apparent increase of ca. 0.6°C in CTS_max_ but the effect disappeared after controlling for random effects and differences in variance among trial types. Likewise, hyperoxia had no effect on CT_max_. Surprisingly, we found no effect of hyperoxia on *Ṁ*_O_2__ during the CTS_max_ trials. Interestingly, although all fish increased their *Ṁ*_O_2__ during the acute warming trials, many did not reach *Ṁ*_O_2_,max_ based on earlier critical swimming speed (*U*_crit_) trials at a benign temperature. Together, our results provide insight into the effects of ecologically relevant levels of oxygen saturation on thermal tolerance in fish.

## Teaser

**Highlighted Article:** Oxygen supersaturation does not notably improve the thermal tolerance or metabolic rate of aerobically exercising or static rainbow trout, suggesting that hyperoxia does not mitigate the effects of elevated water temperatures.

## INTRODUCTION

Increases in average global temperatures and more frequent heatwaves can have significant impacts on aquatic ecosystems, including fish communities (Blasco et al., 2022; O'Reilly et al., 2015; Pörtner and Farrell, 2008; Schulte, 2015). Lakes and streams may be especially vulnerable to warming and thermal variability due in part to the relative isolation of these environments, preventing range shifts, and the fact that freshwater ecosystems are affected by myriad stressors (Reid et al., 2019; Woodward et al., 2010). Shallow freshwater environments are often also inherently variable environments. For example, while oxygen solubility typically decreases as water temperature increases, in shallow aquatic environments, oxygen saturation can vary widely (McArley et al., 2022a). Indeed, hypoxia is a widely recognized global concern for many aquatic ecosystems and can have direct impacts on animal physiology and fitness (Breitburg et al., 2018; Pollock et al., 2007). Conversely, at midday when temperatures peak, localized, transient hyperoxia (oxygen supersaturation >100%) can occur as a result of heightened photosynthetic activity associated with the peak in solar radiation (McArley et al., 2022a).

The effect of naturally occurring midday oxygen supersaturation was recently examined in six species of marine ectotherms from the Red Sea (two fishes and four invertebrates; Giomi et al., 2019). Hyperoxia caused a marked increase in thermal tolerance (LT_50_) of all six species during acute warming trials (Giomi et al., 2019). However, the benefits of hyperoxia in Giomi et al.’s (2019) study are somewhat of an outlier in what is a limited amount of literature on the topic. A more recent study examined the effects of hyperoxia on the critical thermal maximum (CT_max_) of 14 ectotherms from across the globe and found no effect in 10 of 14 species studied, and mixed or weak evidence in the other four species (Raby et al., 2025). McArley et al. (2022a) also compiled data from 10 studies examining the effects of hyperoxia on CT_max_ in 20 species. They found hyperoxia improved thermal tolerance in 9 of 20 species, with the benefits ranging from +0.4°C to +1.8°C (McArley et al., 2022a).

Despite the somewhat mixed results for measures such as thermal tolerance, a study by McArley et al. (2021) did find that hyperoxia can increase the partial pressure of oxygen in arterial blood (*P*a_O_2__). Increased *P*a_O_2__ during exposure to hyperoxia is thought to be the result of the diffusion gradient between water and the blood entering the branchial circulation (McArley et al., 2021). Indeed, in 17 of 18 species examined to date, elevated *P*a_O_2__ occurred under environmental hyperoxia, including in rainbow trout (*Oncorhynchus mykiss*). Elevated *P*a_O_2__ has been detected as soon as 10 min after hyperoxia exposure and can persist as long as the exposure lasts (McArley et al., 2021). Similarly, mixed venous oxygen partial pressure (*P*v_O_2__) was elevated under hyperoxia (200% oxygen saturation) in European perch (*Perca fluviatilis*), suggesting increased oxygen availability to the tissues, which has been proposed to improve thermal tolerance (Ekström et al., 2016). Elevated *P*a_O_2__ is likely to also enable higher maximum rates of whole-animal oxygen consumption. In the widespread small-bodied fish *Galaxias maculatus*, both short- and long-term hyperoxia exposure (150% air saturation) were associated with an increase in maximum metabolic rate (MMR) and aerobic scope of >50% (Skeeles et al., 2022). Similarly, in two intertidal triple fin species, there was an increase in oxygen consumption (*Ṁ*_O_2__) under hyperoxia during thermal ramping at temperatures above 26°C (McArley et al., 2018).

Clarifying whether hyperoxia, which can appear to remove the limits of oxygen supply, can affect upper thermal tolerance is important to determine what mechanism(s) is proximally involved in tolerance to acute warming (Ern et al., 2023). A widely known hypothesis termed oxygen and capacity limited thermal tolerance (OCLTT) proposes that as temperature increases, metabolic oxygen demand increases as well, creating a mismatch between a fish's increased metabolic demand and its ability to deliver oxygen to tissues (Blasco et al., 2022; Pörtner et al., 2017). It does seem plausible that one way in which thermal limits can be reached is when an organism's cardiorespiratory system can no longer meet its cellular oxygen demand (Ern et al., 2023). Thus, examining patterns in oxygen uptake in normoxia versus hyperoxia as fish approach their thermal limits may help inform whether oxygen supply has a central, universal role in upper thermal tolerance in water breathers.

While upper thermal tolerance in fishes is typically assessed in static water via a CT_max_ test (Beitinger and Lutterschmidt, 2011; Desforges et al., 2023; Lutterschmidt and Hutchison, 1997), it can also be assessed while fish are forced to exercise, increasing their demand for oxygen. Recent studies (Blasco et al., 2020, 2022) have introduced an alternative to CT_max_ in which fish are exercised aerobically at 85% of their individually determined critical swimming speed (*U*_crit_) while the water is warmed at a controlled rate (i.e. critical thermal maximum while swimming, CTS_max_). CTS_max_ therefore incorporates the combined demands of sustained exercise and warming, resulting in a metric of thermal tolerance that may be more ecologically relevant than traditional CT_max_. Instead of the endpoint of loss of equilibrium (LOE) commonly used in CT_max_ trials, CTS_max_ trials use the endpoint of swimming failure (the inability to continue swimming against the current). Because CTS_max_ can be evaluated in an intermittent stop-flow swim tunnel respirometer, the rate of oxygen uptake (*Ṁ*_O_2__) by the animal can be measured in concert (Steffensen, 1989). Estimates of thermal tolerance obtained in CTS_max_ experiments are expected to be lower than those obtained during traditional CT_max_ experiments as fish are subjected to acute warming and sustained swimming simultaneously (Blasco et al., 2020).

If a fish's oxygen uptake capacity is a factor regulating its thermal tolerance, and fish have a higher oxygen demand while swimming, then thermal tolerance trials with fish performing aerobic swimming may be ideal conditions under which to assess the role of oxygen limitation. While several studies have examined differences in oxygen consumption and thermal tolerance between CT_max_ and CTS_max_ trials (Blasco et al., 2020, 2022; Nati et al., 2023, 2024; Navarro et al., 2025), none have investigated the effects of hyperoxia on these responses across both trial types. This is particularly relevant given the elevated oxygen demands during CTS_max_ trials and the potential for hyperoxia to meet some of these demands. Thus, we quantified the thermal tolerance of rainbow trout (*O. mykiss*) in CT_max_ and CTS_max_ trials under a hyperoxia treatment (150% oxygen saturation) and a normoxia treatment (100% oxygen saturation). We hypothesized that thermal tolerance would be higher under hyperoxia (cf. normoxia) because of oxygen limitation as fish approach lethal temperatures, and that the difference would be further amplified in fish warmed while swimming aerobically (CTS_max_), given that aerobic work combined with acute heat stress would further increase their cellular demands for O_2_. We also predicted that fish would attain higher *Ṁ*_O_2_ _under hyperoxia (Ekström et al., 2016; Skeeles et al., 2022). The data presented here add to our effort to clarify the role of oxygen limitation in thermal tolerance in water breathers, which may be especially relevant for animals living in shallow, productive aquatic ecosystems that see variability in both temperature and dissolved oxygen.

## MATERIALS AND METHODS

### Ethics approval

Experiments were performed at the Trent University Animal Care Facility from 25 July to 20 December 2023. Animal care and experiments were conducted in accordance with Canadian Council on Animal Care (CCAC) guidelines, with an approved animal use protocol (#28105) from Trent University's Animal Care Committee.

### Animal collection and care

**Fig. 1.**
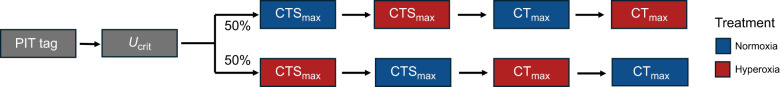
**Schematic diagram of the experimental protocol schedule displaying critical swimming speed and thermal tolerance trials.** Fish (*n*=16) were brought into the lab, PIT tagged, given 1 week to recover prior to experimentation. First, fish were tested in a critical swimming speed (*U*_crit_) trial in a swim tunnel respirometer to determine each fish's individual maximum sustainable swimming speed. *U*_crit_ trials were conducted at 18°C, with fish undergoing a 15 min practice swim and a 30 min rest period before the trial began. Fish then completed two critical thermal maximum trials whilst swimming (CTS_max_ trials) in swim tunnel respirometers, where they were swum at 85% of their *U*_crit_ under hyperoxia (150% dissolved oxygen, DO; red; *n*=15) or normoxia (100% DO; blue; *n*=16), while temperature was increased in stepwise increments from 21°C until fatigue; 50% of the fish completed a trial under hyperoxia, followed by one under normoxia, while the order was reversed for the other 50% of fish. Prior to each CTS_max_ trial, fish were given 15 min to habituate to the swim tunnel while temperature increased from 19 to 21°C. Following the CTS_max_ trials, fish performed two critical thermal maximum (CT_max_) trials in intermittent stop-flow respirometers, one under hyperoxia (red; *n*=11) and one under normoxia (*n*=11), where the trial order alternated in the same manner. Prior to the CT_max_ trials, standard metabolic rate (SMR) was measured overnight at 15°C (holding temperature). The following morning, temperature was increased from 15°C to 21°C, after which CT_max_ trials began, and temperature was increased in stepwise increments, similar to CTS_max_ trials until fish lost equilibrium. Fish were given a minimum of 1 week to recover between trials.

Rainbow trout [*Oncorhynchus mykiss* (Walbaum 1792); *n*=16, mean±s.d. initial fork length 153±40 mm, initial mass 38.9±8.3 g] were obtained from Linwood Acres Fish Hatchery (Campbellcroft, ON, Canada) on 17 July and 3 August 2023. Fish were transported to Trent University in insulated containers filled with continuously aerated water (∼15°C; 30 min by road). Fish were then held in 1500 l tanks equipped with a continuous flow-through system with water drawn from the adjacent Otonabee River that was filtered with a sand filter and ozonation and then chilled. The fish were housed at 14.88±0.45°C (mean±s.d.) for the duration of the study and were allowed to acclimate to the laboratory conditions for 2 weeks prior to experimentation. All fish were also tagged for individual identification upon arrival (Biomark HPT-8 PIT tag). To do so, fish were anaesthetized in an 80 mg l^−1^ concentration of tricaine methane sulfonate (MS222; buffered with 160 mg l^−1^ sodium bicarbonate) after which the PIT tag was inserted above the left pelvic fin using a sterilized 12-gauge needle. Fish were given 1 week to recover from tagging prior to experimentation (Fig. 1). Fish were fed a ration of trout pellets (ca. 0.5% body mass day^−1^) and were fasted for a minimum of 18 h (i.e. overnight) prior to use in experiments to minimize the effects of digestion on metabolism or swimming performance (Chabot et al., 2016; Killen et al., 2021). Each fish was given at least 1 week to recover between experiments (Fig. 1).

### Swimming performance

Swim tunnel respirometry experiments were conducted with a 10 l swim tunnel respirometer (Loligo Systems, Viborg, Denmark; www.loligosystems.com). Prior to experimentation, water velocity in the swim tunnel was established using a calibration curve relating motor setting to the average cross-sectional velocity of the swim tunnel. Solid blocking corrections were not applied as the maximum cross-sectional area of the fish was <10% of the cross-sectional area of the swim tunnel (Bell and Terhune, 1970). Each fish underwent an initial trial to determine its maximum sustainable swimming speed (*U*_crit_; 23 July 2023 to 8 August 2023) prior to the commencement of CTS_max_ trials (9 August 2023 to 4 December 2023). Oxygen consumption (*Ṁ*_O_2__) was measured during all trials (see ‘Oxygen consumption’, below). The swim tunnel was flushed with water from a surrounding water bath, which was supplied with a constant inflow of filtered, chilled and aerated river water, which was thermostatically held at 18.09±0.34°C (mean±s.d.) during each *U*_crit_ trial. First, fish performed a 15 min practice swim at a swimming speed of 45 cm s^−1^ (ca. 3 body lengths s^−1^). This was followed by a 30 min rest period (10 cm s^−1^) to allow fish to acclimate to the swim tunnel, before the *U*_crit_ trial began. For *U*_crit_, the speed was increased to 45 cm s^−1^ over the course of 5 min, followed by progressive speed increments of 10 cm s^−1^ every 20 min until the fish fatigued. Fatigue was determined as the fish's inability to disengage from the rear grid, despite being prompted by minor electric shocks (8 V), light and sound, for a duration of 10 s. Once the fish fatigued, the speed was immediately reduced to 10 cm s^−1^ and the fish was removed from the chamber. The fish was then given 20 min to recover in aerated 18°C water before mass (nearest 0.1 g) and fork length (mm) were taken (Table 1) and the fish was returned to its holding tank.

**Table 1.** Mean (±s.d.) body mass and fork length of fish at the time of each experimental trial

Trial	Mass (g)	Fork length (mm)
PIT tagging	38.9±8.3	153.4±10.1
*U*_crit_	41.8±9.0	156.0±10.4
CTS_max_ normoxia	62.2±17.3	177.3±19.8
CTS_max_ hyperoxia	61.7±17.5	177.0±19.4
CT_max_ normoxia	84.9±19.6	202.5±16.3
CT_max_ hyperoxia	88.2±18.5	204.0±15.4

*U*_crit_, critical swimming speed; CTS_max_, critical thermal maximum while swimming; CT_max_, critical thermal maximum.

### Oxygen consumption

Oxygen was measured throughout all trials using FireSting-O_2_ meters (PyroScience GmbH, Bremen, Germany) equipped with fibre-optic oxygen sensors recording dissolved oxygen (in mg l^−1^) at 1 Hz (with automatic temperature compensation from the Firesting-O_2_ temperature probe). We used intermittent stop-flow respirometry (Steffensen, 1989) whereby the chamber was sealed for 8–15 min during each increment. After each sealed period, the chamber was flushed with aerated water from the surrounding water bath to ensure oxygen saturation in the chamber returned to >95% air saturation before the next *Ṁ*_O_2__ measurement (sealed period). An additional pump was used to recirculate water throughout the water bath to ensure proper mixing of the water throughout the trial (Killen et al., 2021). Oxygen probes were placed inside the mixing circuit of the swim tunnel and calibrated using a two-point calibration procedure (Killen et al., 2021). An upper calibration point [100% dissolved oxygen (DO)] was performed prior to each trial while a lower calibration point (0% DO) was performed every few days. Oxygen readings were adjusted automatically based on the temperature probe placed in the swim tunnel. At the end of each day, the chambers were sealed without a fish and background respiration was measured for 20 min (with the swim tunnel motor set to a moderate speed, ca. 75% of *U*_crit_). The rate of change of oxygen concentration quantified during the background measurement was used to adjust estimates of fish *Ṁ*_O_2_ _from trials conducted that day. After each trial, chambers were drained, rinsed, wiped down and refilled with filtered water to minimize any accumulation of waste or microbes.

### Thermal tolerance

Each fish underwent four thermal tolerance trials under two oxygen treatments: normoxia (100% oxygen saturation) and hyperoxia (150% oxygen saturation). All fish underwent two CTS_max_ trials followed by two CT_max_ trials, with a minimum of 1 week between each trial. Half the fish (*n*=8) underwent CTS_max_ trials under normoxia first, while the order was reversed for the other fish (*n*=8). The same was done for CT_max_ trials, which began 6 days after the final CTS_max_ trials were completed. In both CTS_max_ and CT_max_ trials, the temperature inside the chamber was increased by 1°C every 30 min until the fish reached its endpoint (exhaustion, i.e. failure to continue swimming, or loss of equilibrium, respectively). However, we typically ramped temperature up quickly during the initial part of the 30 min increment (the first ∼5 min) and then kept a more stable temperature during the middle period of each increment when *Ṁ*_O_2__ was measured to minimize temperature change during the *Ṁ*_O_2__ measurement. After each trial, fish were immediately removed from the respirometer and allowed to recover at 20°C for 20 min before mass and fork length were recorded (this measurement of body mass was used for estimates of *Ṁ*_O_2__); they were then returned to the holding tanks.

For CTS_max_, fish were initially placed into the swim tunnel at a speed of 10 cm s^−1^; flow was gradually increased for each fish over the course of 15 min until they reached 85% of *U*_crit_. During this time, temperature was also increased from 19°C to 21°C. Flow speed was then kept at 85% of each fish's individual *U*_crit_ to ensure each fish swam at the same proportion of its swimming capacity and to mitigate effects of inter-individual differences in swimming performance (Blasco et al., 2020, 2022). During the trials, *Ṁ*_O_2_ _was measured by sealing the chamber for a period of 8–15 min depending on the individual fish and temperature increment in order to acquire a sufficient decline in DO to estimate *Ṁ*_O_2_ _without allowing DO to drop below 80% of air saturation. For normoxia trials, the water bath was thoroughly aerated using air stones to ensure oxygen saturation remained above 95% air saturation. For hyperoxia trials, compressed oxygen was manually bubbled into the surrounding bath and regularly adjusted to ensure DO remained at ca. 150% in the water bath. DO was carefully monitored in the water bath throughout using a YSI ProSolo Meter to ensure oxygen saturation remained within 10% of 150%. At the end of the trial, once the fish were removed, the chamber was resealed in order to measure background respiration. Background *Ṁ*_O_2_ _was 10.87±15.81% (mean±s.d.) of the fish's *Ṁ*_O_2_ _for CTS_max_ trials.

For the CT_max_ trials, we used a set of eight 5.6 l polypropylene static intermittent-flow respirometry chambers submerged in a well-aerated 15°C water bath. Fish were placed into the chambers and left overnight to estimate individual standard metabolic rate (SMR) at 15°C. The chambers were cyclically sealed for a period of 13 min and then flushed with water from the surrounding water bath for 17 min to allow DO to return to >95% air saturation using automatic digital timers connected to the flush pumps. FireSting-O_2_ meters were used for these chambers as well, with 10 cm robust oxygen probes inserted into an external recirculation loop for each chamber. Leak tests prior to the first trial confirmed that all chambers were fully sealed when the flush pumps were switched off (with 600 l h^−1^ recirculation pumps still running). The next morning, the temperature of the water bath was increased to 21°C over the course of an hour before the trial began. During the trial, *Ṁ*_O_2_ _was measured by sealing the chambers for a period of 8–13 min during each temperature increment. The endpoint for CT_max_ trials was LOE (Baroudy and Elliott, 1994) as determined by the fish being unable to right itself for at least 5 s. When a fish lost equilibrium, it was removed from the respirometer and allowed to recover for 15 min before being weighed and measured. After the final trial, fish were immediately euthanized with a lethal overdose (250 mg l^−1^) of buffered MS-222 before being weighed and measured. Immediately following the completion of each trial, background respiration was measured in all chambers (mean±s.d. 10.54±12.13% of fish *Ṁ*_O_2__).

### Data analysis and statistics

*U*_crit_ values were calculated using the following equation, where *U*_f_ is the speed of the last fully completed increment (cm s^−1^), *T*_f_ is the duration of time the fish swam at the final increment before it reached fatigue (s), *t* is the length of time at each speed increment (s) and *U*_v_ is the velocity increment (here, 10 cm s^−1^; Kern et al., 2018):

(1) $$U_{\mathrm{crit}}=U_{f}\;+(T_{f}\;t^{-1})\;U_{v}.$$

CTS_max_ and CT_max_ values were calculated in a similar manner to *U*_crit_, by taking the temperature of the last increment the fish completed fully and adding it to the proportion of the final increment the fish completed before it fatigued or experienced LOE (Baroudy and Elliott, 1994; Blasco et al., 2020). Metabolic rate was calculated using the decline in oxygen concentration per minute (mg O_2_ kg^−1^ min^−1^) following guidelines outlined by Killen et al. (2021). Plots of DO from all trials are available from figshare (https://doi.org/10.6084/m9.figshare.31116286). Briefly, fish mass was subtracted from the chamber volume before metabolic rate was calculated in Excel. Background respiration was also measured and removed from metabolic rate measurements. Plots showing raw traces of oxygen and temperature from all trials, and the slopes used to calculate *Ṁ*_O_2__ from those traces, are also available from figshare (https://doi.org/10.6084/m9.figshare.31116286).

All statistical analyses were conducted in R (version 2023.12.0+369, https://www.r-project.org/). As a result of mortalities over the course of the experimental procedure, sample sizes differed for the various trials completed: *U*_crit_ *n*=16, CTS_max_ normoxia *n*=16, CTS_max_ hyperoxia *n*=15, CT_max_ normoxia *n*=11, CT_max_ hyperoxia *n*=11. Paired *t*-tests (paired using animal ID) were used to compare CTS_max_ and CT_max_ values between treatments. A mixed model, with trial type and oxygen treatment as fixed effects and animal ID as a random effect, was also used to determine the effects of treatment and trial type on thermal tolerance. The interaction term between treatment and trial type was insignificant and dropped from the model. To account for differences in residual variance among treatment groups, a variance structure allowing residual variance to differ by treatment was included in the model, which improved model fit. Individual repeatability was quantified using the interclass correlation coefficient (ICC), representing the proportion of total variance attributed to the difference among individuals across thermal tolerance trials. Repeatability was estimated using a linear mixed-effects model with individual identity as a random effect and analyses were conducted using the rptR package in R (Stoffel et al., 2017). For the *Ṁ*_O_2_ _data, generalized additive mixed models (GAMMs) with treatment and temperature as fixed effects and individual ID as a random effect were used to assess the effects of treatment and temperature on metabolic rate. Two GAMMs were used, one for each trial type (CT_max_ and CTS_max_). Interaction terms were insignificant in both cases and dropped from the models. Variance structures were included in both models to account for differences in variance between treatments. A mixed-effects model, with trial type and treatment for fixed effects and individual identification as a random effect, was also used to assess whether there was an effect of trial type or temperature on *Ṁ*_O_2_,peak_ (i.e. maximum *Ṁ*_O_2__ estimated across all temperature increments) of each fish during each trial. Lastly, a linear regression was used to determine whether CTS_max_ values had a relationship with *Ṁ*_O_2_,peak_. Model assumptions (normality, homoscedasticity, independence of residuals and fit of smooth terms) were checked using diagnostic plots. The type I error rate (α) was set to 0.05.

## RESULTS

### Swimming performance

During the *U*_crit_ trials, fish exhibited steady swimming until they approached their maximum swimming speed, after which they exhibited burst and glide behaviour (indicative of anaerobic swimming) before they fatigued. During CTS_max_ trials, fish exhibited steady swimming until they reached a certain temperature, ranging from ca. 23 to 26°C, where they then also exhibited unsteady swimming and burst glide behaviour, which ultimately led to fatigue. The mean CTS_max_ across all trials was 26.70°C (range: 23.75–28.40°C). All fish recovered from the CTS_max_ trials, and no fish exhibited loss of equilibrium during these trials. Four of 15 fish that underwent CT_max_ trials failed to recover (i.e. did not regain equilibrium); the mean CT_max_ across all fish was 29.02°C (range: 28.41–29.71°C).

### Thermal tolerance

**Fig. 2.**
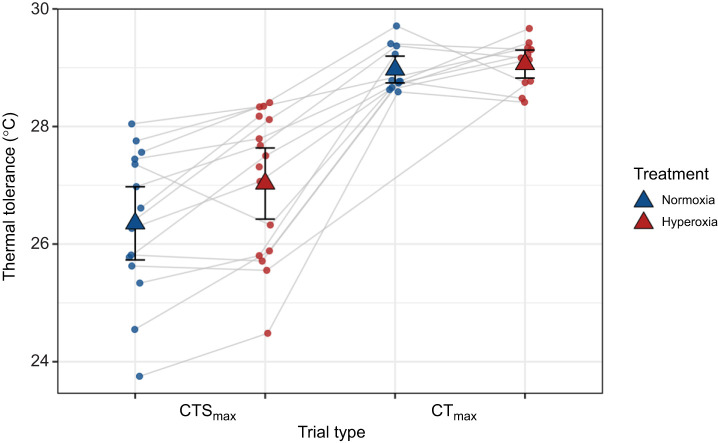
**Temperature at which rainbow trout reached their CTS_max_ and CT_max_ under normoxia and hyperoxia.** Large triangles represent means and error bars represent s.e.m.; small circles represent individual animals and grey lines link data for individual animals (CTS_max_ *n*=15–16, CT_max_ *n*=9–11). Statistical differences were assessed using linear mixed-effects models (*P*=0.18). Note that in the experiment, the order in which fish were exposed to the hyperoxia versus normoxia treatment was randomly assigned (fish were split into two different groups).

We found weak support for an effect of hyperoxia on thermal tolerance in rainbow trout. There was a tendency for higher CTS_max_ under hyperoxia than in normoxia (Fig. 2; paired *t*-test, difference in means of 0.66°C; *P*=0.004). However, in our overall model (Table 2) controlling for animal ID and including a variance structure to account for differences in variance between trial types, this effect was not statistically significant (overall hyperoxia effect term: *P*=0.26; interaction term between treatment and trial type was similarly non-significant and dropped from the final model; *P*=0.18). Additionally, inclusion of oxygen treatment had no effect on model fit (ΔAIC<1, log-likelihood test *P*=0.24). Trial type had a clear effect on thermal tolerance, as expected, whereby CT_max_ was markedly higher (2.33±0.22°C, *P*<0.001) and less variable (Levene's test, *P*<0.001) than CTS_max_. Indeed, CTS_max_ values spanned a range of 23.75 to 28.41°C, whereas CT_max_ spanned a much smaller range of 28.41 to 29.71°C. Thermal tolerance across the experiments was moderately repeatable at the individual level (*R*^2^=0.49, 95% confidence interval CI=0.159, 0.735).

**Table 2.** Parameters and their statistical significance for a linear mixed-effects model comparing thermal tolerance of rainbow trout as a function of trial type (CTS_max_ and CT_max_) and oxygen treatment (hyperoxia and normoxia)

Parameter	Value±s.e.	d.f.	*t*	*P*	Variance	s.d.
Fixed effects						
Intercept	28.95±0.12	35	236.24	<0.001		
Trial type (CTS_max_)	−2.33±0.22	35	−10.63	<0.001		
Treatment (Hyperoxia)	0.16±0.14	35	1.14	0.26		
Random effects						
Effect						
ID					0.06	0.24
Residual					1.23	1.11

Fixed effects were trial type and oxygen treatment, random effect was individual animal ID.

### Oxygen consumption

**Fig. 3.**
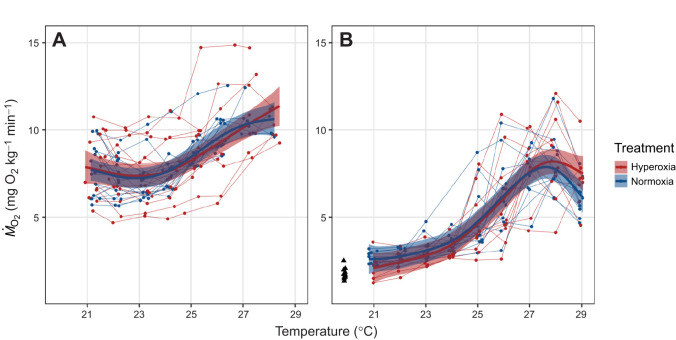
**Generalized additive mixed models of oxygen uptake (*Ṁ*_O_2__) of rainbow trout during CTS_max_ and CT_max_ trials under normoxia and hyperoxia.** (A) CTS_max_ (*P*<0.001) and (B) CT_max_ (*P*<0.001) trials under normoxia (blue) and hyperoxia (red). Thick lines represent fitted smooth terms from a generalized additive mixed model and shaded areas represent 95% confidence intervals. Small circles represent *Ṁ*_O_2__ measurements for individual fish and thin lines represent repeated measures for individual fish (CTS_max_ trials under hyperoxia *n*=15 and for normoxia *n*=16; CT_max_ trials under both oxygen treatments *n*=11). Black triangles represent SMR taken at 15°C (*n*=13).

We found strong support for an effect of temperature on *Ṁ*_O_2__ but weak support for an effect of hyperoxia. In CTS_max_ trials, the effect of temperature was significant and non-linear for both the hyperoxic (GAMM: effective degrees of freedom edf=3.21, *f*=13.36, *P*<0.001) and normoxic (edf=4.35, *f*=20.04, *P*<0.001) treatments. This model also controlled for animal ID and included a variance structure to account for differences between trial types. For both treatments, *Ṁ*_O_2__ remained stable until ca. 24°C when *Ṁ*_O_2__ increased with further increases in temperature (Fig. 3). However, the effects of oxygen saturation on *Ṁ*_O_2__ were marginal when comparing model fits with and without oxygen saturation as a factor (log-likelihood test: *P*=0.038, ΔAIC=10.6), with no clear separation between the treatments at any temperature (Fig. 3A). ΔAIC also favoured a simpler model, indicating weak evidence that treatment had a significant effect on *Ṁ*_O_2__ (Table 3). Additionally, the simpler model explained slightly more variance (*R*^2^=0.369) than did the model with oxygen treatment (*R*^2^=0.359).

**Table 3.** Model comparison results for generalized additive mixed models (GAMMs) describing the effects of temperature and oxygen saturation on metabolic rate (*Ṁ*_O_2__) during CTS_max_ trials

Model	Formula	d.f.	AIC	ΔAIC
M3	*Ṁ*_O_2__∼s(Temp) + Random(ID) + Var(Treatment) + AR	8	583.9	0
M2	*Ṁ*_O_2__∼s(Temp, by Treatment) + Random(ID)+Var(Treatment) + AR	10	594.4	10.5527
M5	*Ṁ*_O_2__∼s(Temp, by Treatment) + Random(ID) + AR	9	601.1	17.1795
M4	*Ṁ*_O_2__∼s(Temp, by Treatment) + Random(ID) + Var(Treatment)	8	676	92.0759
M1	*Ṁ*_O_2__∼s(Temp, by Treatment) + Random(ID)	7	688.7	104.786

Models were compared using Akaike's information criterion (AIC); ΔAIC values indicate the difference from the best-supported model. Temp refers to water temperature; ID indicates individual fish identity; Treatment refers to oxygen saturation treatment; Var(Treatment) represents a variance structure by treatment; and AR represents the correlation structure.

In CT_max_ trials, the effects of temperature on *Ṁ*_O_2_ _were similarly non-linear under hyperoxia (edf=4.59, *f*=34.7, *P*<0.001) and normoxia (edf=5.34, *f*=27.61, *P*<0.001). Once ca. 28°C was reached, *Ṁ*_O_2__ began to decline with temperature – this decline appeared to be slightly more pronounced under normoxia compared with hyperoxia (Fig. 3B). However, the best-fit model (*P*=0.007, ΔAIC=14) did not include oxygen treatment (Table 4). The model for *Ṁ*_O_2__ in the CT_max_ trials accounted for a moderately high amount of variation (*R*^2^=0.687). SMR was also calculated for each fish prior to CT_max_ trials with a mean of 1.75±0.08 mg O_2_ kg^−1^ min^−1^. Aerobic scope was not estimated because SMR was measured at a different temperature from that for *Ṁ*_O_2_,peak_, and at a different time (and therefore body size) from that for *U*_crit_.

**Table 4.** Model comparison results for GAMMs describing the effects of temperature and oxygen saturation on *Ṁ*_O_2__ during CT_max_ trials

Model	Formula	d.f.	AIC	ΔAIC
M8	*Ṁ*_O_2__∼s(Temp) + Random(ID) + Var(Treatment) + AR	8	680.6411065	0
M10	*Ṁ*_O_2__∼s(Temp, by Treatment) + Random(ID) + AR	9	694.2278103	13.5867038
M7	*Ṁ*_O_2__∼s(Temp, by Treatment) + Random(ID) + Var(Treatment) + AR	10	694.6309036	13.9897971
M6	*Ṁ*_O_2__∼s(Temp, by Treatment) + Random(ID)	7	705.2820676	24.6409611
M9	*Ṁ*_O_2__∼s(Temp, by Treatment) + Random(ID) + Var(Treatment)	8	705.301804	24.6606975

Models were compared using AIC; ΔAIC values indicate the difference from the best-supported model. Temp refers to water temperature; ID indicates individual fish identity; Treatment refers to oxygen saturation treatment; Var(Treatment) represents a variance structure by treatment; and AR represents the correlation structure.

**Fig. 4.**
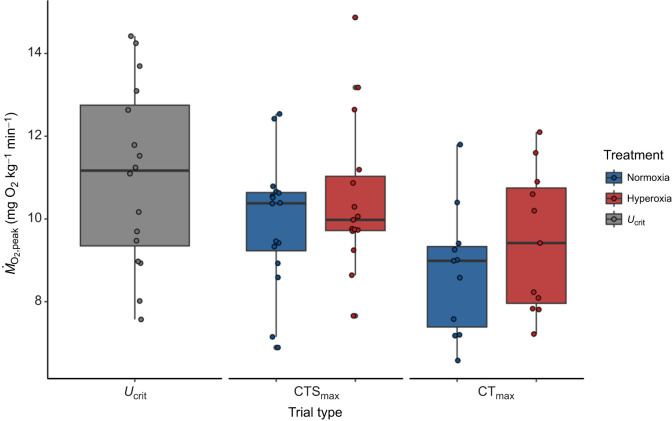
**Peak metabolic rate (*Ṁ*_O_2_,peak_) of rainbow trout across *U*_crit_, CTS_max_ and CT_max_ trials.** CTS_max_ (*n*=15 hyperoxia, *n*=16 normoxia) and CT_max_ (*n*=11) trials were conducted under normoxia (blue) and hyperoxia (red). The *U*_crit_ (*n*=16) trial was only conducted once under 100% DO. Small circles represent individual animals. Box plots show the median (horizontal line), upper and lower quartiles (box limits) and inter-quartile range (whiskers). Statistical differences were assessed using a linear mixed-effects model with fish ID as a random effect (*P*=0.012).

Mean *Ṁ*_O_2_,peak_ was highest during the *U*_crit_ trial (11.68±0.57 mg O_2_ kg^−1^ min^−1^; *P*=0.012; Fig. 4). *Ṁ*_O_2_,peak_ during the *U*_crit_ trials was considered a ‘true’ measurement of maximum metabolic rate as it was measured when fish were exercising maximally. However, for *n*=10 fish, *Ṁ*_O_2__ peaked at a higher level during one of their thermal tolerance trials, albeit these peaks occurred at a higher temperature (typically >25°C) than the *U*_crit_ trial (18°C). *Ṁ*_O_2_,peak_ did differ significantly between CTS_max_ and CT_max_ trials (*P*=0.026), but not between hyperoxia and normoxia treatments in either trial type (linear mixed-effects model; *P*=0.185).

**Fig. 5.**
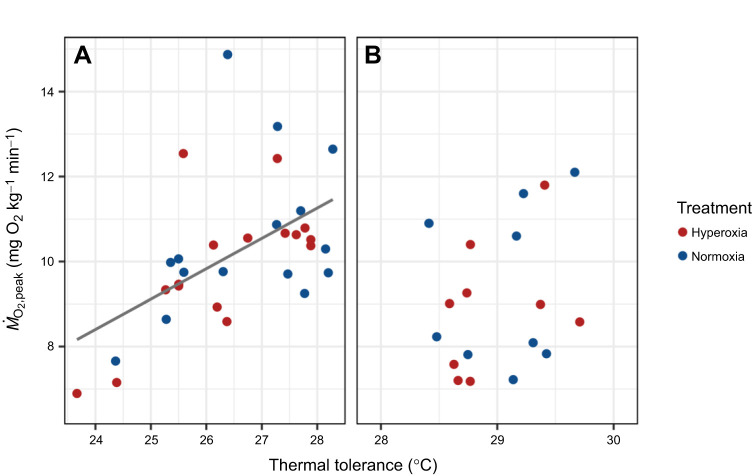
**Linear regression of the relationship between CTS_max_ or CT_max_ and *Ṁ*_O_2_, peak_ of rainbow trout under normoxia and hyperoxia.** (A) CTS_max_ (*n*=16) and (B) CT_max_ (*n*=9) under normoxia (blue) and hyperoxia (red). There was no statistically significant relationship for CT_max_; for CTS_max_, the relationship was significant and therefore the regression line is shown (slope=0.7149, intercept=−8.7578, *R*^2^=0.286, *P*=0.0019).

There was a positive correlation between *Ṁ*_O_2_,peak_ and CTS_max_ (Fig. 5). Within the CTS_max_ trials conducted in this study, an individual's *Ṁ*_O_2_,peak_ had a modest ability to explain inter-individual variation in thermal tolerance (linear regression: *P*=0.0019, *R*^2^=0.286; Fig. 5A). This relationship did not occur in the CT_max_ trials (*P*=0.239, *R*^2^=0.085; Fig. 5B).

## DISCUSSION

### Effects of hyperoxia on thermal tolerance and metabolic rate

As predicted, we found that thermal tolerance during aerobic exercise (measured by CTS_max_) had a tendency for a slight increase under hyperoxia, although the effect was small and unclear. Overall, while not statistically meaningful, the trend was directionally consistent with predictions of the OCLTT hypothesis. The same small effect did not occur in CT_max_ trials, similar to most of the existing data on fishes (whereby there is little or no effect of hyperoxia on CT_max_; McArley et al., 2021; Raby et al., 2025). This may indicate that oxygen availability contributes more strongly to thermal tolerance under conditions of elevated metabolic demand. However, some previous studies have found clearer positive effects of hyperoxia on thermal tolerance, suggesting that oxygen limitation may play a role in regulating the thermal tolerance of some water breathers but not others (Giomi et al., 2019; Jutfelt et al., 2018; McArley et al., 2021; Raby et al., 2025). Here, we present the first data on the effects of hyperoxia on thermal tolerance during aerobic exercise; further work will be needed to assess whether CTS_max_ is affected by hyperoxia more widely than is CT_max_, or whether its effects are similarly variable and species specific. As hyperoxia may play a stronger role in regulating thermal tolerance in fish with lower thermal limits, potentially because these species are limited by oxygen at comparatively lower temperatures during warming (McArley et al., 2021), it will be useful to quantify the effects of hyperoxia on thermal tolerance in CTS_max_ across more species.

We predicted that *Ṁ*_O_2_ _would increase under hyperoxia compared with normoxia in both trial types because of an increased ability to take up oxygen as a result of a stronger diffusion gradient across the gills. This would relate to the mechanism behind a potential increase in thermal tolerance predicted by the OCLTT hypothesis, which proposes that limitations in oxygen supply contribute to thermal tolerance limits. However, we found no significant effect of oxygen treatment on *Ṁ*_O_2__, despite *Ṁ*_O_2_ _appearing to be directionally higher under hyperoxia, except that *Ṁ*_O_2__ was more variable under hyperoxia (among individuals) than in normoxia, particularly in the CTS_max_ trials. Instead, only water temperature had a significant effect on *Ṁ*_O_2_ _during thermal ramping across both trial types. Patterns of oxygen uptake remaining similar under hyperoxia and normoxia indicate that factors beyond oxygen limitation were determinants of thermal tolerance (i.e. not supporting the OCLTT hypothesis). While other studies have observed an increase in either *Ṁ*_O_2_ _or *Ṁ*_O_2_,peak_ under hyperoxia (McArley et al., 2022a,b; Polymeropoulos et al., 2019), no such increase occurred in this study. The decline in *Ṁ*_O_2_ _at the highest temperatures in the CT_max_ trials (across both hyperoxia and normoxia treatments) may indicate that there was a physiological threshold past which cell and/or tissue function was impaired, and the addition of oxygen was not enough to offset the costs of these elevated temperatures (Morrison et al., 2020; Reid and Ricciardi, 2022; Schulte, 2014). The mechanism behind fatigue in CTS_max_ experiments may relate to the capacity for oxygen uptake under thermal stress during exercise, as fish demonstrated that they could maintain this speed (85% of *U*_crit_) without fatiguing under constant, non-stressful temperatures during the *U*_crit_ trials (Blasco et al., 2022; McArley et al., 2022b). McArley and colleagues (2022b) found that the mechanism behind increased *Ṁ*_O_2_ _of rainbow trout in static CT_max_ trials was gill diffusion limitation, which was relieved under hyperoxia. It remains unclear why hyperoxia did not improve thermal tolerance in the CT_max_ trials in this study; however, there is relatively little evidence supporting the hypothesis that thermal tolerance is causally linked to oxygen limitation in most fishes.

We did find that *Ṁ*_O_2_,peak_ was higher in fish that reached higher temperatures in CTS_max_ trials, suggesting that inter-individual variation in aerobic performance was correlated with thermal tolerance. This relationship was consistent with a previous study that found that *Ṁ*_O_2_,peak_ was positively correlated with CTS_max_ in all populations investigated, including captive European seabass (*Dicentrarchus labrax*) and Nile tilapia (*Oreochromis niloticus*), as well as wild-caught European seabass and gilthead seabream (*Sparus aurata*) (Navarro et al., 2025). This relationship was not observed in our (static) CT_max_ trials, though it is important to note that CT_max_ values comprised a much smaller range (28.41–29.71°C) than did CTS_max_ values (23.75–28.41°C), making it quantitatively more difficult to tease apart individual differences. We have no evidence to support a causal link between elevated *Ṁ*_O_2_,peak_ and thermal tolerance, particularly given that *Ṁ*_O_2_,peak_ did not necessarily occur at the highest temperature, immediately before fish fatigued. Therefore, in our view, these data provide only weak support to the notion that higher oxygen uptake can improve thermal tolerance in fish, as suggested in some studies (Ekström et al., 2016; McArley et al., 2018, 2021), but not others (Sandrelli et al., 2024). However, as *Ṁ*_O_2_ _did not increase under hyperoxia in this study, it is possible that an increased ability to take up oxygen may improve thermal tolerance in fish, but that oxygen supersaturation may not always increase a fish's ability to take up oxygen. This is contradictory to results of other studies that have found *Ṁ*_O_2_,max_ increases under hyperoxia, including in rainbow trout (Ekström et al., 2016; McArley et al., 2018, 2021; Skeeles et al., 2022). However, here we did not focus on quantifying an effect of hyperoxia on *Ṁ*_O_2_,max_, which would have required separate *U*_crit_ (or *U*_max_) swimming tests in normoxia and hyperoxia (we only ran these trials under normoxia) given that most fish apparently did not reach their *Ṁ*_O_2_,max_ during thermal ramping in the CTS_max_ trials. A similar pattern has previously been observed where fish did not reach the upper extreme of their cardiorespiratory capacity during CTS_max_ trials (Navarro et al., 2025).

We found that mean CT_max_ was approximately 2°C higher than mean CTS_max_. This difference is consistent with other studies and indicates that fatigue during CTS_max_ trials is a more ‘sensitive’ metric for measuring thermal tolerance in fish compared with LOE under CT_max_ (Blasco et al., 2020, 2022; Nati et al., 2023, 2024; Navarro et al., 2025). Interestingly, we did find that thermal tolerance was repeatable at the individual animal level across both CTS_max_ and CT_max_ trials with varying oxygen tensions, suggesting that individuals with higher thermal tolerance during CTS_max_ also had higher thermal tolerance during CT_max_. While thermal tolerance has been found to be moderately repeatable within individuals for CT_max_ trials (Chasse et al., 2025; Desforges et al., 2023; Morgan et al., 2018), to our knowledge no other studies have examined whether this extends across different measures of thermal tolerance. This consistency across contexts is important, as it suggests that the physiological mechanisms that determine thermal tolerance may be maintained across different contexts, which has implications for the ecological significance and potential heritability of these traits. Nevertheless, it is possible that these two metrics reflect fundamentally different physiological pathways leading to ‘failure’ (i.e. the endpoint) (Jutfelt et al., 2024). The differences in activity levels and range of endpoint temperatures across the two trial types may support this point; a fish that is exercising close to its *U*_crit_ while temperature increases is in a different state from a non-swimming fish in a static respirometer. In either case, CTS_max_ is a useful metric of thermal tolerance. Indeed, CTS_max_ may be particularly relevant for highly active species or for fish exhibiting behaviours where they may be regularly required to swim aerobically, such as foraging, migration or avoiding predation (Blasco et al., 2020; Desforges et al., 2023; McKenzie et al., 2021; Nati et al., 2023, 2024; Navarro et al., 2025).

Understanding the relationship between CT_max_ and CTS_max_ can provide further insight into mechanisms that regulate thermal tolerance in fish and should therefore be explored in other species. We found that *Ṁ*_O_2_,peak_ was lower in CT_max_ than in CTS_max_ trials (including at the same temperatures), as observed in previous studies (Nati et al., 2023). This suggests either that fish were not oxygen limited during CT_max_ trials or that they were unable to elevate *Ṁ*_O_2_ _any further while confined in a static respirometer, perhaps partly due to a lack of ram ventilation (McArley et al., 2022b; Steffensen, 1985). In any case, the data here underlie the fact that oxygen limitation is not a universal determinant of thermal tolerance (Ekström et al., 2019, 2023, 2025; Eliason and Anttila, 2017; Ern et al., 2023). Other physiological pathways besides oxygen limitation likely played some role in the variation in thermal tolerance observed here; exercise is generally required to elicit the highest possible oxygen uptake values (MMR; Rees et al., 2024), and thus *Ṁ*_O_2_,peak_ would be expected to be higher during CTS_max_ trials. However, we expected hyperoxia to increase *Ṁ*_O_2_ _and *Ṁ*_O_2_,peak_ during both CTS_max_ and CT_max_ trials. Its failure to do so suggests this species or context was an imperfect test of oxygen limitation as a mechanism of thermal tolerance despite a previous study that reported higher *Ṁ*_O_2_,max_ under hyperoxia in rainbow trout (McArley et al., 2022a).

### Conclusion and ecological relevance

This is the first study to examine the effects of hyperoxia on thermal tolerance in fish that are aerobically exercising. We found little evidence that hyperoxia improved thermal tolerance. The potential for hyperoxia to increase thermal tolerance in freshwater fish may have ecological implications in some contexts, particularly in shallow or highly productive environments where localized hyperoxia occurs naturally (McArley et al., 2021). If the OCLTT held true, hyperoxic conditions could mitigate the effects of elevated temperatures by improving aerobic performance (Skeeles et al., 2022). Wild populations of rainbow trout and other salmonid species are going to be increasingly exposed to temperatures that approach their thermal limits. Many rainbow trout populations near the limit of their range, such as those in Southern California, are regularly exposed to stressful temperatures (>25°C), and experience reduced thermal safety margins (the difference between their upper thermal tolerance and the upper temperatures they occupy in nature) (Desforges et al., 2023; Sunday et al., 2014). While some populations may be locally adapted to higher temperatures, there is little opportunity to behaviourally thermoregulate in some ecosystems if conditions become too stressful (Dressler et al., 2023). The fact that CTS_max_ is lower than CT_max_ suggests fish can exhibit physiological failure at temperatures lower than those which directly threaten their survival (Blasco et al., 2020). For example, if a fish forages more, and therefore exercises more, to maintain metabolic performance under elevated temperatures, it may be at increased risk of approaching its CTS_max_ (as opposed to CT_max_). This highlights the value of quantifying a variety of metrics of thermal tolerance (i.e. beyond CT_max_), as different endpoints may reflect different but complementary physiological pathways, which may further inform species-specific thermal risk assessments (Blasco et al., 2020, 2022; Desforges et al., 2023).
